# T_2_-Weighted Whole-Brain Intracranial Vessel Wall Imaging at 3 Tesla With Cerebrospinal Fluid Suppression

**DOI:** 10.3389/fnins.2021.665076

**Published:** 2021-06-25

**Authors:** Lei Zhang, Yanjie Zhu, Yulong Qi, Liwen Wan, Lijie Ren, Yi Zhu, Na Zhang, Dong Liang, Ye Li, Hairong Zheng, Xin Liu

**Affiliations:** ^1^Paul C. Lauterbur Research Center for Biomedical Imaging, Shenzhen Institutes of Advanced Technology, Chinese Academy of Sciences, Shenzhen, China; ^2^Department of Radiology, Peking University Shenzhen Hospital, Shenzhen, China; ^3^Department of Neurology, Shenzhen No. 2 People’s Hospital, Shenzhen, China

**Keywords:** intracranial vessel wall imaging, atherosclerosis, stroke, SPACE, T_2_IR

## Abstract

**Background:**

T_2_-weighted (T_2_w) intracranial vessel wall imaging (IVWI) provides good contrast to differentiate intracranial vasculopathies and discriminate various important plaque components. However, the strong cerebrospinal fluid (CSF) signal in T_2_w images interferes with depicting the intracranial vessel wall. In this study, we propose a T_2_-prepared sequence for whole-brain IVWI at 3T with CSF suppression.

**Methods:**

A preparation module that combines T_2_ preparation and inversion recovery (T_2_IR) was used to suppress the CSF signal and was incorporated into the commercial three-dimensional (3D) turbo spin echo sequence-Sampling Perfection with Application optimized Contrast using different flip angle Evolution (SPACE). This new technique (hereafter called T_2_IR-SPACE) was evaluated on nine healthy volunteers and compared with two other commonly used 3D T_2_-weighted sequences: T_2_w-SPACE and FLAIR-SPACE (FLAIR: fluid-attenuated inversion recovery). The signal-to-noise ratios (SNRs) of the vessel wall (VW) and CSF and contrast-to-noise ratios (CNRs) between them were measured and compared among these three T_2_-weighted sequences. Subjective wall visualization of the three T_2_-weighted sequences was scored blindly and independently by two radiologists using a four-point scale followed by inter-rater reproducibility analysis. A pilot study of four stroke patients was performed to preliminarily evaluate the diagnostic value of this new sequence, which was compared with two conventional T_2_-weighted sequences.

**Results:**

T_2_IR-SPACE had the highest CNR (11.01 ± 6.75) compared with FLAIR-SPACE (4.49 ± 3.15; *p* < 0.001) and T_2_w-SPACE (−56.16 ± 18.58; *p* < 0.001). The subjective wall visualization score of T_2_IR-SPACE was higher than those of FLAIR-SPACE and T_2_w-SPACE (T_2_IR-SPACE: 2.35 ± 0.59; FLAIR-SPACE: 0.52 ± 0.54; T_2_w-SPACE: 1.67 ± 0.58); the two radiologists’ scores showed excellent agreement (ICC = 0.883).

**Conclusion:**

The T_2_IR preparation module markedly suppressed the CSF signal without much SNR loss of the other tissues (i.e., vessel wall, white matter, and gray matter) compared with the IR pulse. Our results suggest that T_2_IR-SPACE is a potential alternative T_2_-weighted sequence for assessing intracranial vascular diseases.

## Introduction

Intracranial atherosclerotic disease is a leading cause of ischemic stroke worldwide, particularly in the Asian population (Kim and Johnston, 2011; Qureshi and Caplan, 2014). High-resolution magnetic resonance (MR) intracranial vessel wall imaging (IVWI) has been reported as a promising technique allowing direct visualization of intracranial atherosclerotic plaques (Swartz et al., 2009; Qiao et al., 2011; Zhu et al., 2016; Mandell et al., 2017; Young et al., 2019). The characterization of intracranial vessel walls using MR imaging requires suppressing the MR signal arising from luminal blood and cerebrospinal fluid (CSF) (Qiao et al., 2011; van der Kolk et al., 2013; Dieleman et al., 2014; Mandell et al., 2017; Young et al., 2019), helping to delineate both the inner and outer walls of the vessels. Early studies mainly focused on T_1_-weighted IVWI because of its ability to reveal vessel wall abnormalities with (i.e., atherosclerotic plaque) or without contrast agents (i.e., intraplaque hemorrhage), and it can also help classify intracranial vasculopathy (i.e., vasculitis) (Qiao et al., 2011; van der Kolk et al., 2013; Dieleman et al., 2014; Zhang et al., 2015; Mandell et al., 2017; Young et al., 2019; Jia et al., 2020).

Recently, studies have shown that T_2_-weighted IVWI has the potential to identify intracranial plaque components and classify plaque types (van der Kolk et al., 2015; Harteveld et al., 2016; Jiang et al., 2016). For example, T_2_-weighted IVWI allows the identification of lipid cores and fibrous cap ruptures (Turan et al., 2013; Chung et al., 2014). Xu et al. (2010) reported that a hyperintense band adjacent to the lumen on T_2_-weighted images might suggest a fibrous cap. Ryu et al. (2009) reported that the foci of T_2_ hyperintensity within plaques were more frequently observed in symptomatic stenosis than in asymptomatic stenosis. Additionally, T_2_-weighted IVWI can be used as a complementary tool in multi-contrast vessel wall imaging for classifying intracranial vasculopathy (Mossa-Basha et al., 2015, 2016, 2017) and detecting atherosclerotic lesions that are not visible on magnetic resonance angiography (MRA) (Li et al., 2009).

Although T_2_-weighted IVWI shows great potential in clinical use, its bright CSF signal makes the outer boundary of the intracranial vessel wall indistinguishable and may lead to estimation bias in vessel wall thickness. Fluid-attenuated inversion recovery (FLAIR) imaging has been applied to three-dimensional (3D) T_2_-weighted IVWI to suppress the CSF signal (Turan et al., 2013). However, using inversion recovery requires a long inversion time for adequate CSF suppression and causes a significant deficiency in the signal-to-noise ratio (SNR). Another technique that combines T_2_ preparation and an inversion recovery pulse (referred to as T_2_IR) was developed to suppress background tissue for flow-independent peripheral angiography (Brittain et al., 1997); its advantages of an improved SNR and reduced T_1_-weighting make it suitable for various applications (i.e., cardiac MRI, vessel wall imaging, and cerebral blood mapping) (Brittain et al., 1995; Mugler et al., 2000; Wong et al., 2001; Rooney et al., 2007; Busse et al., 2008; Liu et al., 2010, 2017; Visser et al., 2010; Xie et al., 2010; Mugler, 2014; Zhao et al., 2016; Zhang D.F. et al., 2017; Qi et al., 2018; Qin et al., 2019; Zhang et al., 2019; Zi et al., 2020). The T_2_IR preparation module was used to achieve a submillimeter volumetric FLAIR sequence in an ultra-high field system (Visser et al., 2010).

In the present study, we combined the T_2_IR preparation module with 3D variable-flip-angle TSE- Sampling Perfection with Application optimized Contrast using different flip angle Evolution (SPACE) acquisition, called T_2_IR-SPACE, and achieved high resolution IVWI at 3.0T. The performance of the new sequence was assessed in a simulation study and an *in vivo* study in healthy volunteers and patients. The preliminary work for this study was partially reported in Zhang et al. (2019).

## Materials and Methods

### Pulse Sequence

**FIGURE 1:**
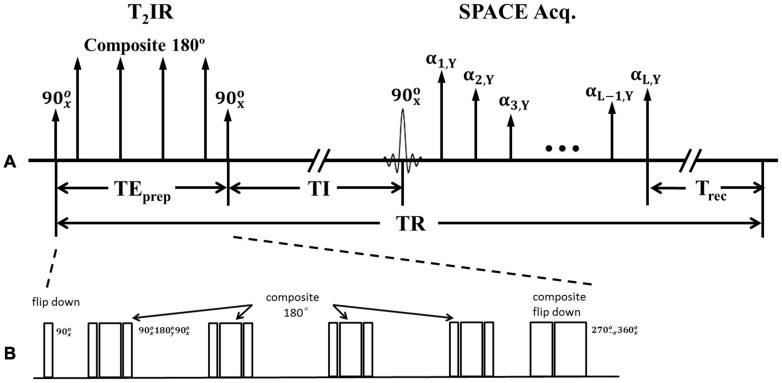
**(A)** Schematic of the T_2_IR preparation module followed by SPACE acquisition. **(B)** Zoom in of T_2_IR preparation module.

The proposed T_2_IR-SPACE sequence comprises two parts (Figure 1): a T_2_IR preparation module (Brittain et al., 1997) and T_2_-weighted SPACE acquisition sampling (Mugler et al., 2000; Mugler, 2014) at the null point of the CSF signal.

Figure 1B illustrates the timing diagram of the T_2_IR preparation module used in the present study. This module is designed according to the Carr-Purcell Malcolm Levitt (MLEV) method (Brittain et al., 1995). First, the longitudinal magnetization is excited by a $90_{\text{x}}^{\text{o}}$ radiofrequency (RF) pulse. Next, four composite refocus pulses ($90_{\text{x}}^{\text{o}}-180_{\text{y}}^{\text{o}}-90_{\text{x}}^{\text{o}}$) are applied, with the phases alternated to minimize the adverse effects of B_1_ and B_0_ field inhomogeneities. Finally, a composite $90_{\text{x}}^{\text{o}}$ ($270_{-\text{x}}^{\text{o}}-360_{\text{x}}^{\text{o}}$) pulse tips the T_2_-prepared transverse magnetization down to the −*z* axis. The pulse is designed following the composite pulse ($270_{\text{x}}^{\text{o}}-360_{-\text{x}}^{\text{o}}$) of $-90_{\text{x}}^{\text{o}}$ (Brittain et al., 1995) but with an opposite phase to tip the magnetization downward. The duration of the T_2_IR module, TE_prep_, weights the longitudinal magnetization by −e^–TEprep/T2^. A spoiling gradient is applied to dephase all the remaining transverse magnetizations (not shown in Figure 1). The SPACE acquisition is delayed by the time of inversion (TI) from the last 90° pulse at the null point of the CSF signal. All the pulses used are hard pulses, and the durations of the 90°, 180°, 270°, and 360° RF pulses are 0.5, 1, 1.5, and 2 ms, respectively.

### Simulations

Bloch simulations (Busse et al., 2008; Zhang et al., 2015) were performed to investigate the signal behaviors of the intracranial vessel wall and CSF in the T_2_IR-SPACE sequence. The simulation parameters were as follows: TR/TE = 2500/92 ms; echo spacing (ESP) = 4.4 ms; echo train length (ETL) = 77; TE_prep_ = 200 ms; and TI = 950 ms. The T_1_ and T_2_ values were 4300 and 2200 ms for CSF (Rooney et al., 2007; Liu et al., 2017), and 1200 and 50 ms for the vessel wall (Xie et al., 2010; Qi et al., 2018). The simulation was implemented and performed in MATLAB version 2010 (MathWorks, Natick, MA, United States). The optimization of the parameters of T_2_IR module (TE_prep_ and TI) is provided in the Supporting Material (Supplementary Figure 1).

The signal evolutions of the vessel wall and CSF in conventional 3D T_2_-weighted vessel wall imaging (T_2_w-SPACE) and 3D T_2_-weighted vessel wall imaging with a FLAIR preparation pulse to suppress CSF (FLAIR-SPACE) were also simulated and compared with the proposed T_2_IR-SPACE sequence. The imaging parameters were adjusted to achieve the same spatial resolution and spatial coverage in the same scan time for all three sequences. The simulation parameters for T_2_w-SPACE were the same as those for T_2_IR-SPACE (Supplementary Figure 2 shows the optimization of T_2_w-SPACE). For FLAIR-SPACE, the simulation parameters were TR/TE = 6250/345 ms, ESP = 4.4 ms, ETL = 195, and TI = 2100 ms. The recently developed techniques, such as delay alternating with nutation for tailored excitation (DANTE) prepared T_2_w-SPACE and AntiDrive were also compared with our proposed T_2_IR-SPACE (Yang et al., 2016; Fan et al., 2017; Viessmann et al., 2017; Zhang L. et al., 2017). The Parameters for the DANTE module were: flip angle = 8°, number of pulses = 150, maximum gradient (in x, y, and z directions) = 20 mT/m, interpulse duration = 1.5 ms.

### *In vivo* Experiments

All the experiments were performed using a 3T clinical whole-body MR system (TIM TRIO, Siemens, Erlangen) equipped with a 32-channel head coil. Nine healthy volunteers (three female; aged 24–61 years; mean age: 44.9 years) without known cerebrovascular disease were recruited for the volunteer study. Four patients (one female; aged 33–52 years) with symptoms of stroke and a diagnosis of intracranial arterial stenosis based on earlier MR angiography or computed tomography angiography were recruited for the pilot study. The patients were recruited during initial hospitalization within 30 days of symptom onset. Two more volunteers (both females, aged 62 and 29 years) were recruited to compare DANTE prepared T_2_w-SPACE and AntiDrive with T_2_IR-SPACE. Both studies were approved by the institutional review board, and informed consent forms were signed by all the participants before MR imaging.

For each volunteer, IVWI using whole-brain coverage was performed using T_2_IR-SPACE, T_2_w-SPACE, and FLAIR-SPACE. The imaging parameters were the same as those in the simulation and were summarized in Table 1. All the sequences were performed in the sagittal orientation. The fat suppression technique used in this study was composed of a spectral-selective pulse and spoiling gradients. GRAPPA was used to accelerate the scan time. The total scan time was 11 min 40 s for each of the three sequences.

**TABLE 1:** Imaging parameters of the sequences evaluated in the study.

	**T_2_IR-SPACE**	**FLAIR-SPACE**	**T_2_w-SPACE**
TR/TE (ms)	2500/92	6250/345	2500/123
TE_prep_/TI (ms)	200/950	…/2100	…/…
Echo train length	77	195	77
**Common parameters**			
Matrix size	288 × 288 × 224		
FOV (mm)	170 × 170 × 134.4		
Slice partial Fourier	5/8		
Bandwidth (Hz/pixel)	579		
GRAPPA/ref. lines	2/24		
Fat suppression	Yes		
Flip angle mode	T_2_ var		
Voxel size (mm)	0.6 isotropic		
Scan time	11 min 40 s		

In the patient study, three scans (T_2_IR-SPACE, T_2_w-SPACE, and FLAIR-SPACE, respectively) were conducted for two patients. The other two patients underwent only T_2_IR-SPACE and T_2_w-SPACE scans because they could not endure the long scan times. The imaging parameters were the same as those in the volunteer study.

### Image Analysis

Qualitative image analysis was performed at a workstation (Syngo MultiModality Workplace, Siemens Healthcare, Germany) by two experienced radiologists (Q.Y.L. and Z.Y. with over 10 and 7 years of experience in neurovascular imaging, respectively) independently. The 3D image sets of T_2_IR-SPACE, FLAIR-SPACE and T_2_w-SPACE were presented to the two radiologists individually in a random order, with imaging information being blinded. The intracranial vascular beds were divided into three segments for assessment: Segment 1 included the M1–2 segments of the middle cerebral artery (MCA); Segment 2 included the basilar artery (BA) and V3–4 segments of VA; Segment 3 included C4–7 segments of ICA. Image quality was assessed using a four-point scale: 0 (poor), more than 50% of the vessel walls were invisible; 1 (acceptable), more than 50% of the walls were visible, but with noticeable blurring or limited SNR and CNR between the vessel wall and CSF; 2 (good), vessel walls were continuously visible but were slight blurred; 3 (excellent), vessel walls were clearly depicted with good SNR, CNR and sharpness. Scores >1 were regarded as diagnostic.

Quantitative analysis was performed at the following three vessel segments surrounded by CSF with different flow rate: the M1 segment of the MCA of both the left and right sides, BA, and internal carotid artery cavernous segment (C4, ICA) of both the left and right sides. T_2_IR-SPACE, FLAIR-SPACE and T_2_w-SPACE images were co-registered on the workstation using 3D image fusion functionality (Syngo Fusion, Siemens, Germany). 2D cross-sectional wall images were reconstructed by an experienced MRI scientist (Z.N.) using multiplanar reconstruction for each arterial segment; five vessel segments were reconstructed for each subject. Care was taken to ensure location matching among different scans. The SNR of the vessel wall and adjacent CSF and the contrast-to-noise ratio (CNR) between them were measured using region-of-interest (ROI) analysis. Based on the aforementioned 2D images, the ROI was manually prescribed on the images where the vessel walls were clearly visualized in all three sequences, and the mean signal intensities (S) of these sequences were obtained. The SNR is defined as SNR = S/σ, where S is the mean signal intensity of a certain tissue (VW or CSF) and σ is the noise measured as the standard deviation from an artifact-free air region of the nasal cavity. The CNR between the VW and CSF is defined as CNR = SNR_VW_-SNR_CSF_.

Statistical analyses were performed by using SPSS software (version 19.0; Chicago, IL, United States). The intra-reader correlation coefficient (ICC) was obtained from a two-way random model. The ICC value was interpreted as excellent, good, fair, and poor when it was between 0.75 and 1, between 0.6 and 0.74, between 0.4 and 0.59, and less than 0.4, respectively. Paired two-tailed Wilcoxon signed-rank test was performed on the data sets to determine the significance of the differences. The significance level was set at *p* < 0.05/2 = 0.025 (Bonferroni correction). The data were presented as means ± standard deviation.

## Results

### Simulations

**FIGURE 2:**
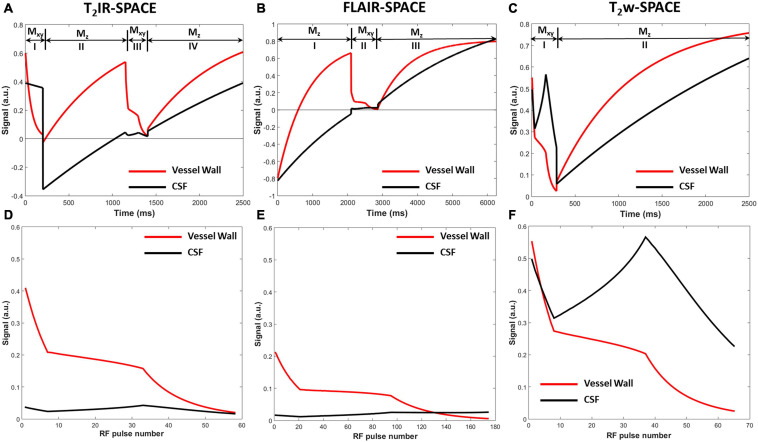
Signal evolutions of the vessel wall and CSF for **(A)** T_2_IR-SPACE, **(C)** FLAIR-SPACE, and **(E)** T_2_w-SPACE sequences within one TR using Bloch simulation. Signal evolutions of transverse magnetization during the acquisition for **(B)** T_2_IR-SPACE, **(D)** FLAIR-SPACE, and **(F)** T_2_w-SPACE.

The simulated signal evolutions within one TR were plotted after ten repetitions of the pulse sequence until the signal evolutions reached the steady-state for subsequent repetitions. The signal evolution for T_2_IR-SPACE (Figure 2A) comprises four parts: (I) transverse magnetization (M_xy_) modulated by T_2_ decay during the T_2_IR module, in which, the M_xy_ of CSF decreases slightly because of its long T_2_ value and the M_xy_ of vessel wall is close to zero at the end of the T_2_IR module; (II) a second $90_{\text{x}}^{\text{o}}$ pulse tipping the transverse magnetization to the negative longitudinal axis, and the M_z_ recovering from the −*z* axis during TI; (III) M_xy_ during the SPACE acquisition that performs around the null point of CSF; and (IV) recovery of the M_z_ during T_rec_. The signal evolution for FLAIR-SPACE (Figure 2C) comprises three parts: (I) recovery of the M_z_ from the −*z* axis after the inversion recovery pulse; (II) M_xy_ during the SPACE acquisition around the null point of CSF; and (III) recovery of M_z_ during the remainder of the TR. T_2_w-SPACE signal evolution (Figure 2E) has two parts: (I) M_xy_ during the SPACE acquisition and (II) recovery of M_z_ during the remaining time of TR. Figures 2B,E,F are the magnified blocks from Figures 2A,B,E, showing the M_xy_ during the SPACE acquisition in T_2_IR-SPACE, FLAIR-SPACE, and T_2_w-SPACE, respectively. The CSF signals were well suppressed in both T_2_IR-SPACE and FLAIR-SPACE (both < 0.05). The signal intensity of the vessel wall in T_2_IR-SPACE (∼0.2) was almost twice that in FLAIR-SPACE (∼0.1). Although T_2_w-SPACE had the highest vessel wall signal (∼0.25) among these three sequences, the unsuppressed CSF signal (∼0.5) resulted in a low contrast between the vessel wall and CSF.

### *In vivo* Experiments

**FIGURE 3:**
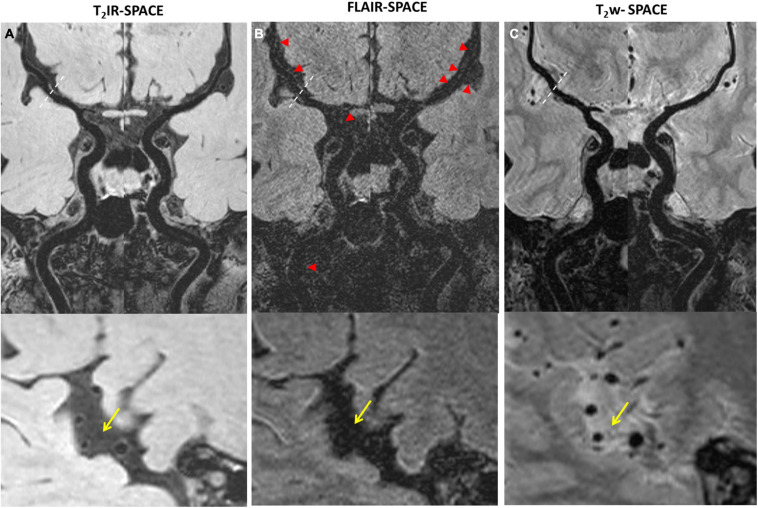
Comparison among **(A)** T_2_IR-SPACE, **(B)** FLAIR-SPACE, and **(C)** T_2_w-SPACE images in a healthy 60-year-old volunteer. Curved multi-planar reconstruction images of both the left and right sides, starting from the internal carotid artery (ICA) and continuing up to the M2 segment of the middle cerebral artery (MCA), were generated for visual comparison. Bottom images show the zoomed-in image to better show the inner/outer vessel wall for each sequence, CSF area are labeled by the arrows. **(A)** The vessel walls are clearly visualized at all segments of intracranial arteries on T_2_IR-SPACE. **(B)** The SNR is low on FLAIR-SPACE, and most of the intracranial vessel wall cannot be delineated (indicated by the red arrowheads). **(C)** For T_2_w-SPACE, delineating the outer boundary of the intracranial artery wall is challenging.

The MR scans were successfully acquired in all the participants with adequate or excellent image quality. As expected, the CSF signal was effectively suppressed in all T_2_IR-SPACE images while the signal of other tissues maintained a high level. The qualitative image analysis results are summarized in Table 2. T_2_IR SPACE showed better overall image quality when visualizing the intracranial vessel wall (reader 1, T_2_IR-SPACE: 2.35 ± 0.59; FLAIR-SPACE: 0.52 ± 0.54; T_2_w-SPACE: 1.671 ± 0.58). The inter-reader reliability was 0.883 (0.794–0.93; *p* < 0.0001). Representative images are shown in Figure 3. The vessel walls were clearly visualized at all segments of intracranial arteries on T_2_IR-SPACE images, while they were almost invisible at most segments on FLAIR-SPACE images because of their low SNRs (labeled by red arrows). Additionally, the intracranial vessel wall could not be differentiated in T_2_w-SPACE images because of the high CSF signal.

**TABLE 2:** Comparison of the vessel wall visualization quality among T_2_IR, FLAIR, and T_2_w based on a four-point scale (0, poor; 1, fair; 2, good; and 3, excellent).

		**T_2_IR-SPACE**	**FLAIR-SPACE**		**T_2_w-SPACE**	
		**Radiology scores (mean ± SD)**	**Radiology scores (mean ± SD)**	***p*-value: vs. T_2_IR**	**Radiology scores (mean ± SD)**	***p*-value: vs. T_2_IR**
Reader 1	MCA	2.78 ± 0.44	0.67 ± 0.50	0.006	1.67 ± 0.50	0.008
	BA	2.22 ± 0.67	0.11 ± 0.33	0.006	1.67 ± 0.50	0.059
	ICA	2.56 ± 0.53	0.89 ± 0.33	0.006	2.11 ± 0.33	0.102
	Overall	2.52 ± 0.58	0.56 ± 0.51	<0.001	1.81 ± 0.48	<0.001
Reader 2	MCA	2.11 ± 0.33	0.22 ± 0.44	0.006	1.22 ± 0.44	0.011
	BA	2.00 ± 0.50	0.11 ± 0.33	0.006	1.44 ± 0.73	0.059
	ICA	2.44 ± 0.73	1.11 ± 0.33	0.014	1.89 ± 0.60	0.132
	Overall	2.19 ± 0.56	0.48 ± 0.58	<0.001	1.51 ± 0.64	0.001
Mean		2.35 ± 0.59	0.52 ± 0.54	<0.001	1.67 ± 0.58	0.001

The quantitative results are summarized in Table 3. T_2_IR-SPACE showed the best image contrast between the vessel wall (VW) and CSF (CNR: 11.01 ± 6.75) among the three sequences. As expected, the SNRs of the VW and CSF from T_2_w-SPACE were the highest (VW: 50.45 ± 18.50; CSF: 106.61 ± 7.70), but the high CSF signal made delineating the outer boundary of the intracranial vessel wall challenging. The CSF signal was effectively suppressed in both T_2_IR-SPACE and FLAIR-SPACE, but the T_2_IR-SPACE had a much higher vessel wall signal (20.57 ± 6.07 vs. 9.40 ± 3.06; *p* < 0.001), resulting in a higher CNR between the VW and CSF (11.01 ± 6.75 vs. 4.49 ± 3.15; *p* < 0.001). The CNRs approximately agreed with the Bloch simulation predictions shown in Figure 2.

**TABLE 3:** SNR and CNR measurement results.

	**T_2_IR-SPACE**	**FLAIR-SPACE**		**T_2_w-SPACE**	
	**Measurement (mean ± SD)**	**Measurement (mean ± SD)**	***p*-value: vs. T_2_IR**	**Measurement (mean ± SD)**	***p*-value: vs. T_2_IR**
**SNR: VW**	20.57 ± 6.07	9.40 ± 3.06	<0.001	50.45 ± 18.50	<0.001
**SNR: CSF**	9.55 ± 1.87	4.91 ± 1.87	<0.001	106.61 ± 7.70	<0.001
**CNR: VW-CSF**	11.01 ± 6.75	4.49 ± 3.15	<0.001	−56.16 ± 18.58	<0.001

**FIGURE 4:**
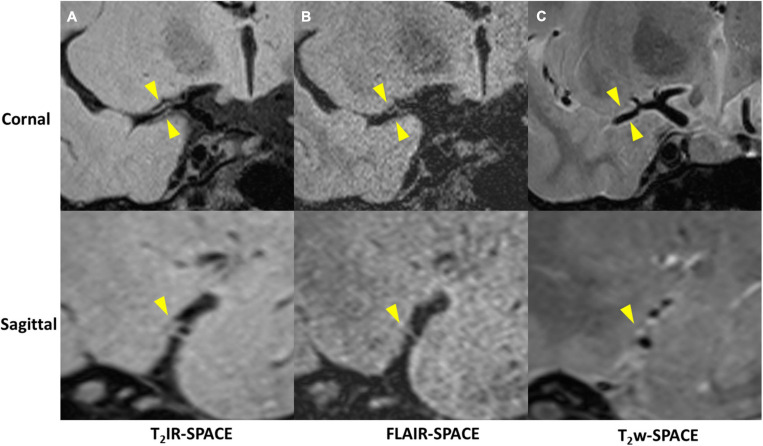
A 33-year-old female patient diagnosed with vasculitis. **(A)** T_2_IR-SPACE, **(B)** FLAIR-SPACE, and **(C)** T_2_w-SPACE images. Top: coronal view of the right MCA; Bottom: sagittal view of the thickened vessel wall pointed by the arrowheads. Among the three T_2_w sequences, only T_2_IR-SPACE depicted concentric wall thickening on the M1 segment of the right MCA; the vessel wall was not clear on FLAIR-SPACE, and most parts of the vessel wall were missing; the vessel wall on T_2_-weighted SPACE imaging appeared normal.

**FIGURE 5:**
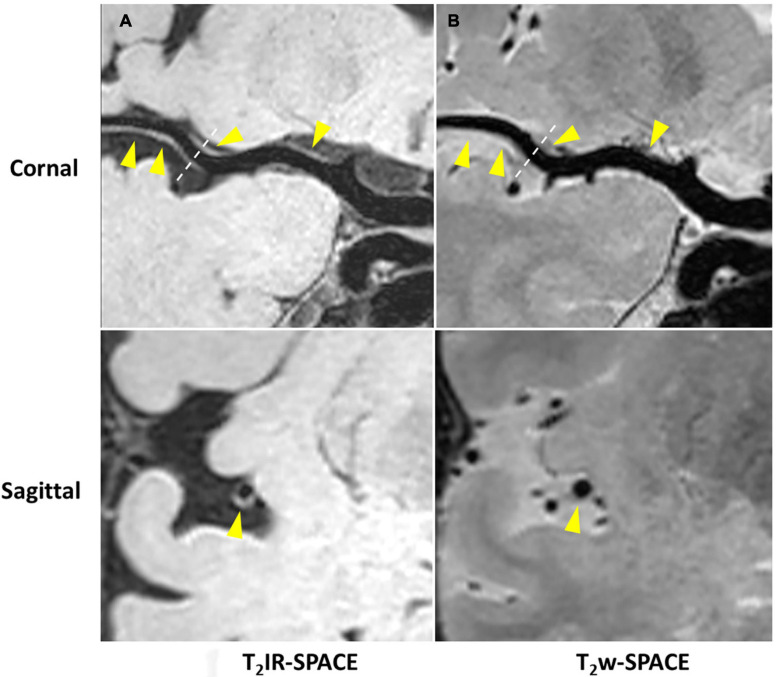
Curved MPR of **(A)** T_2_IR-SPACE and **(B)** T_2_w-SPACE from a 46-year-old male patient. Top: coronal view of the right MCA; Bottom: sagittal view of artery at the location of the dashed line; Wall thickening was depicted only in T_2_IR-SPACE (labeled by the arrowhead). The vessel appeared normal in T_2_w-SPACE.

**FIGURE 6:**
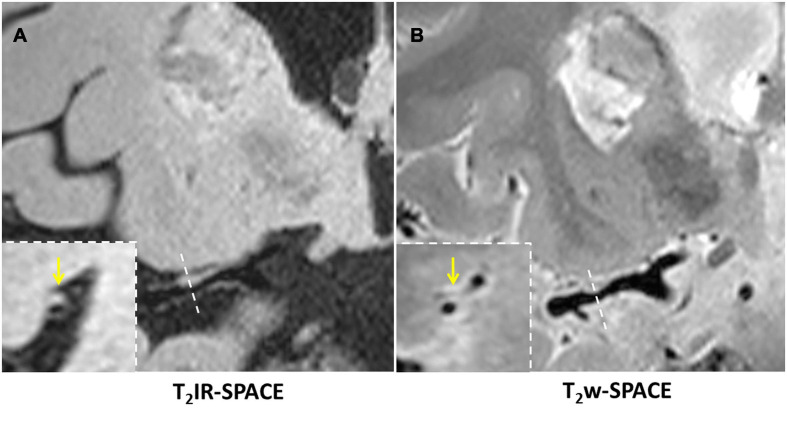
In a 45-year-old male patient with ischemic stroke, **(A)** reconstructed T_2_IR-SPACE image and **(B)** T_2_w-SPACE identified a plaque at the M1 segment of the right MCA. However, the cross-sectional view (bottom dashed insert) showed eccentric wall thickening only on T_2_IR-SPACE because of the signal suppression of surrounding CSF. The outer boundary of the eccentric plaque was not visible on T_2_w-SPACE. The yellow arrow means the eccentric wall thickening.

Imaging was successfully performed in all four patients. Among the four patients, three patients were found to have atherosclerosis plaques at the location of stenosis. One patient (female, 33 years old) was diagnosed as probable vasculitis (Figure 4), concentric wall thickening at MCA was more conspicuous on the T_2_IR-SPACE image than on the FLAIR-SPACE and T_2_w-SPACE images. Figures 5, 6 show the patients with atherosclerosis, in which wall thickening at the M2 segment of MCA was depicted only in T_2_IR-SPACE imaging (Figure 5).

**FIGURE 7:**
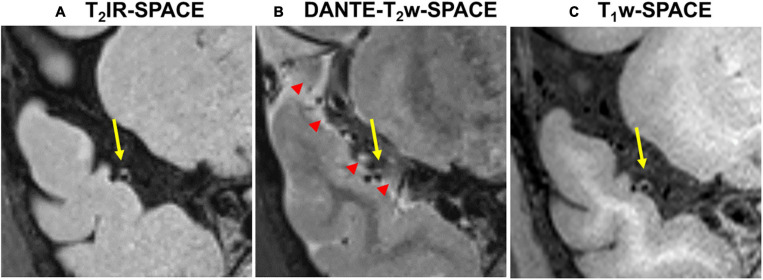
Comparison between **(A)** T_2_IR-SPACE and **(B)** DANTE prepared T_2_w-SPACE in a 62-year-old subject. **(C)** T_1_w-SPACE was scanned as a reference standard. The yellow arrows indicate the wall thickening which is visualized in T_2_IR-SPACE and T_1_w-SPACE. The red arrowheads indicate the heterogeneous CSF signal caused by the DANTE module.

**FIGURE 8:**
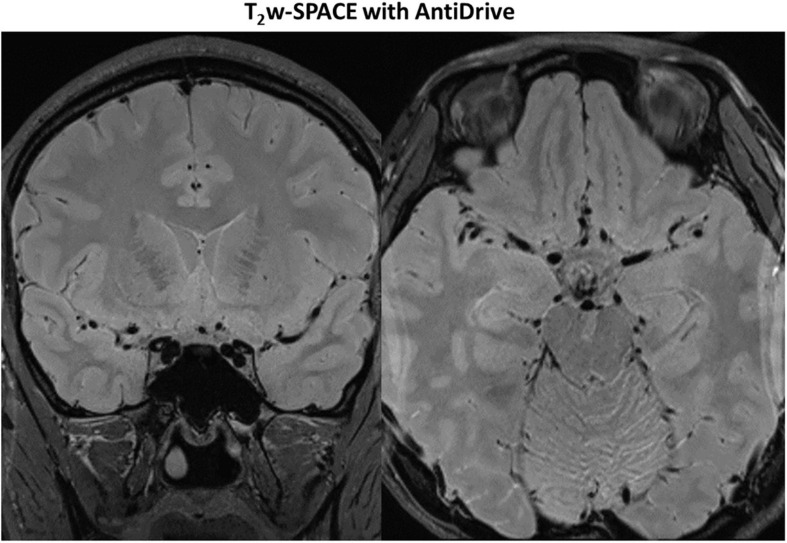
Representative images of T_2_w-SPACE with AntiDrive from a healthy subject. The CSF is not well suppressed and the intracranial vessel wall is not visible.

Figure 7 shows example images from a 62-years-old subject acquired with T_2_IR-SPACE and DANTE prepared T_2_w-SPACE, where T_1_w-SPACE was also scanned as a reference standard. Wall thickening was detected at the M2 segment of the right MCA in T_2_IR-SPACE (yellow arrow). This lesion was confirmed in T_1_w-SPACE but could not be visualized in DANTE-SPACE. We performed T_2_w-SPACE with AntiDrive in another healthy subject. The result is shown in Figure 8, illustrating that the CSF signal is not well suppressed and the intracranial vessel wall is not visible.

## Discussion

T_2_IR-SPACE achieved high spatial resolution, large spatial coverage and, more importantly, remarkable CSF suppression and enhanced WM-CSF CNR. As shown in our preliminary results, T_2_IR-SPACE greatly improves the ability of conventional T_2_w-SPACE to differentiate vessel walls from CSF and is a potential alternative to T_2_w-SPACE for assessing intracranial vascular diseases.

The subjective assessment results showed that T_2_IR-SPACE had better intracranial wall visualization than the other two sequences in all vessel wall segments (Table 2). The subjective mean scores of T_2_IR-SPACE were significantly higher than those of FLAIR-SPACE in all segments. This is because the IR pulse significantly reduced the SNR of the overall image and most parts of the intracranial vessel wall were missing in FLAIR-SPACE images, as demonstrated in both volunteer subjects and patient subjects (Figures 3, 4). Moreover, the radiologist scores of T_2_IR-SPACE were significantly higher than those of T_2_w-SPACE in the MCA and BA segments. This is because in the two regions, and the CSF signal in T_2_-weighted images was high, the outer boundary of the intracranial vessel wall was difficult to differentiate from the surrounding CSF (Figures 3–6), interfering with the accurate diagnosis by the radiologist. In the ICA segment, the radiologist score of T_2_IR-SPACE showed little improvement without statistical significance compared with T_2_w-SPACE because the intracranial vessel wall at the ICA region was not surrounded by the CSF fluid, so the high CSF signal would not influence the vessel wall visualization. Our proposed T_2_IR-SPACE technique compensates for the imperfection of the above two existing approaches. It suppresses the CSF signal without much signal loss of other tissues, and the vessel wall can be clearly visualized at all segments of intracranial arteries in T_2_IR-SPACE images (Figures 3–6).

Previous studies have demonstrated that patients with symptomatic MCA stenosis have larger wall area, plaque area and remodeling index than asymptomatic patients (Xu et al., 2010; Zhang D.F. et al., 2017; Zhao et al., 2016). A hyperintensity band adjacent to the lumen was often observed on T_2_-weighted images, which was assumed to represent the fibrous cap (Xu et al., 2010; Mossa-Basha et al., 2015). In these studies, the MCA-CSF interface was used to manually trace the vessel area. The high CSF signal in T_2_-weighted MRI images makes it difficult to distinguish the outer boundary of the intracranial vessel wall and results in inaccurate measurements of wall area and plaque area. T_2_IR-SPACE suppresses CSF uniformly in whole-brain coverage and allows clear visualization of the interface between the MCA and CSF, which helps to characterize the features of intracranial plaques more accurately and stratify stroke risk in patients with atherosclerotic disease.

Recently, DANTE prepared SPACE and SPACE with AntiDrive have gained popularity in IVWI due to their efficient CSF suppression and superior SNR efficiency (Yang et al., 2016; Fan et al., 2017; Viessmann et al., 2017; Zhang L. et al., 2017). We compared these techniques with our proposed T_2_IR-SPACE in a preliminary volunteer study. The DANTE module suppresses the CSF well around the circle of Wills but varies at distal (M2 or beyond) segments of the MCA or adjacent to the brain parenchyma (Figure 7, red arrowheads), because CSF fluid velocity varies in those regions and DANTE is a velocity-sensitive module, thus resulting in a heterogeneous CSF signal and interfering with accurate diagnosis by the radiologist. The T_2_IR module provides robust CSF suppression regardless of the flow velocity, because this module relies on physical properties (T1 and T2) of CSF rather than the velocity of CSF. Hence, this approach is insensitive to slow flow and flow direction. In T_2_w-SPACE with AntiDrive, the CSF signal is not well suppressed and the intracranial vessel wall is not visible. This is because the TR (2500 ms) is relatively long in T_2_w-SPACE, although the AntiDrive pulse inverts the M_z_ of CSF to the negative longitudinal axis at the end of the echo train, the CSF still recovers to a relatively high value during the long T_rec_. However, in T_1_w-SPACE, CSF signal is already low enough and lower than the brain parenchyma due to the short TR, and the application of AntiDrive pulse would further reduce the CSF signal and improve the T1 contrast. This is the reason why AntiDrive pulse is widely used in T_1_-weighted IVWI.

In this study, a composite 90° pulse was used instead of a hard pulse to tip the magnetization down with better immunity to B_0_ field inhomogeneity. However, the composite pulse has a narrower bandwidth with limited RF amplitude when implemented on a human scanner and is thus sensitive to B_1_ field inhomogeneity.

This study has several limitations. One limitation of the technique is the long scan time as result of the long TR required for CSF nulling. This can not be easily compensated for by e.g., choosing a longer ETL as this would negatively impact the effective resolution of the scan. We however feel that the robustness of CSF suppression justifies the use of the sequence. Furthermore, there have efforts already using compressed sensing for IVWI for T_1_-weighting imaging (Zhu et al., 2018; Jia et al., 2020) and such strategies might be used in T_2_-weighting imaging to enable shortening of the scan time, thereby improving the clinical feasibility of our sequence. Second, we performed simulations and found that T_2_IR-SPACE was affected by partial T_1_-weighting next to the desired T_2_-weighting, due to the short TI time (Supplementary Figure 3). We will optimize the parameters of T_2_IR-SPACE to reduce the T_1_-weighting in future studies. Third, the number of patients involved in this study was small. However, the present study was designed to demonstrate the feasibility and potential of T_2_IR-SPACE in IVWI. Separate clinical studies involving a large patient cohort are needed to evaluate the clinical relevance for the diagnosis of intracranial vascular disease.

## Conclusion

In the present study, we developed a new whole-brain T_2_-weighted intracranial arterial wall imaging sequence with CSF suppression. The T_2_IR preparation module suppresses the CSF signal remarkably without much SNR loss of in other tissues (i.e., vessel wall, white matter, and gray matter) compared with the IR pulse. Our results suggest that T_2_IR-SPACE is a potential alternative T_2_-weighted sequence for assessing intracranial vascular diseases.

## Data Availability Statement

The datasets used and analyzed during this study are available from the corresponding author on reasonable request and filling out NEL-Consent redirecting to “Zenodo” with the doi: https://doi.org/10.5281/zenodo.4145224.

## Ethics Statement

The studies involving human participants were reviewed and approved by Institutional Review Board of Shenzhen Institute of Advanced Technology, Chinese Academy of Sciences; Institutional Review Board of Shenzhen Second People’s Hospital. The patients/participants provided their written informed consent to participate in this study.

## Author Contributions

LZ and YaZ designed the sequence and experiments and drafted the manuscript. LZ performed the Bloch equation simulations. YQ and YiZ performed the subjective assessment. LW contributed to data collection. LR recruited the patients. NZ and LW measured the SNR and CNR. XL and YL contributed to the study design and helped revised the manuscript critically. XL and HZ conceived the study design and provided supervision of the whole project and critical review of the manuscript. All authors read and approved the final manuscript.

## Conflict of Interest

The authors declare that the research was conducted in the absence of any commercial or financial relationships that could be construed as a potential conflict of interest.

## Supplementary Material

Supplementary Figure 1 Simulated T_2_IR-SPACE signal intensity of **(A)** vessel wall, **(B)** CSF, and **(C)** contrast between the vessel wall and CSF as a function of TI and TE_prep_.

Supplementary Figure 2 T_2_w-SPACE images with different TRs in a healthy subject.

Supplementary Figure 3 Simulation results of T_2_IR-SPACE from two hypothetical tissues with the same T2 value and different T1 values **(a,b)**; the same T1 value and different T2 values **(c,d)**.
