# Supplementary material for: T2-Weighted Whole-Brain Intracranial Vessel Wall Imaging at 3 Tesla With Cerebrospinal Fluid Suppression
Source: Front Neurosci. 2021 Jun 25;15:665076. doi: 10.3389/fnins.2021.665076 (PMC8267868; doi:10.3389/fnins.2021.665076)
Supplement: Supplementary Figure 1 — Simulated T2IR-SPACE signal intensity of (A) vessel wall, (B) CSF, and (C) contrast between the vessel wall and CSF as a function of TI and TEprep. [file Table_1.DOCX]

**Supporting Material**

**Optimization of the parameters of T_2_IR-SPACE.**

The parameters of T_2_IR preparation (TE_prep_ and TI) were optimized through simulation. We plotted the signal intensities of the vessel wall and CSF and the contrast between them as a function of TE_prep_ and TI, and the results are shown in Supporting Figure 1. The signal of the vessel wall increases as TE_prep_/TI increases; however, the contrast between the vessel wall and CSF (defined as CNR = vessel wall - CSF) increases as TI increases and then decreases as TI further increases. The contrast peaks at approximately TE_pre_/TI=200/950 ms (supporting Figure 1c, red dot) were the parameters used in this study.


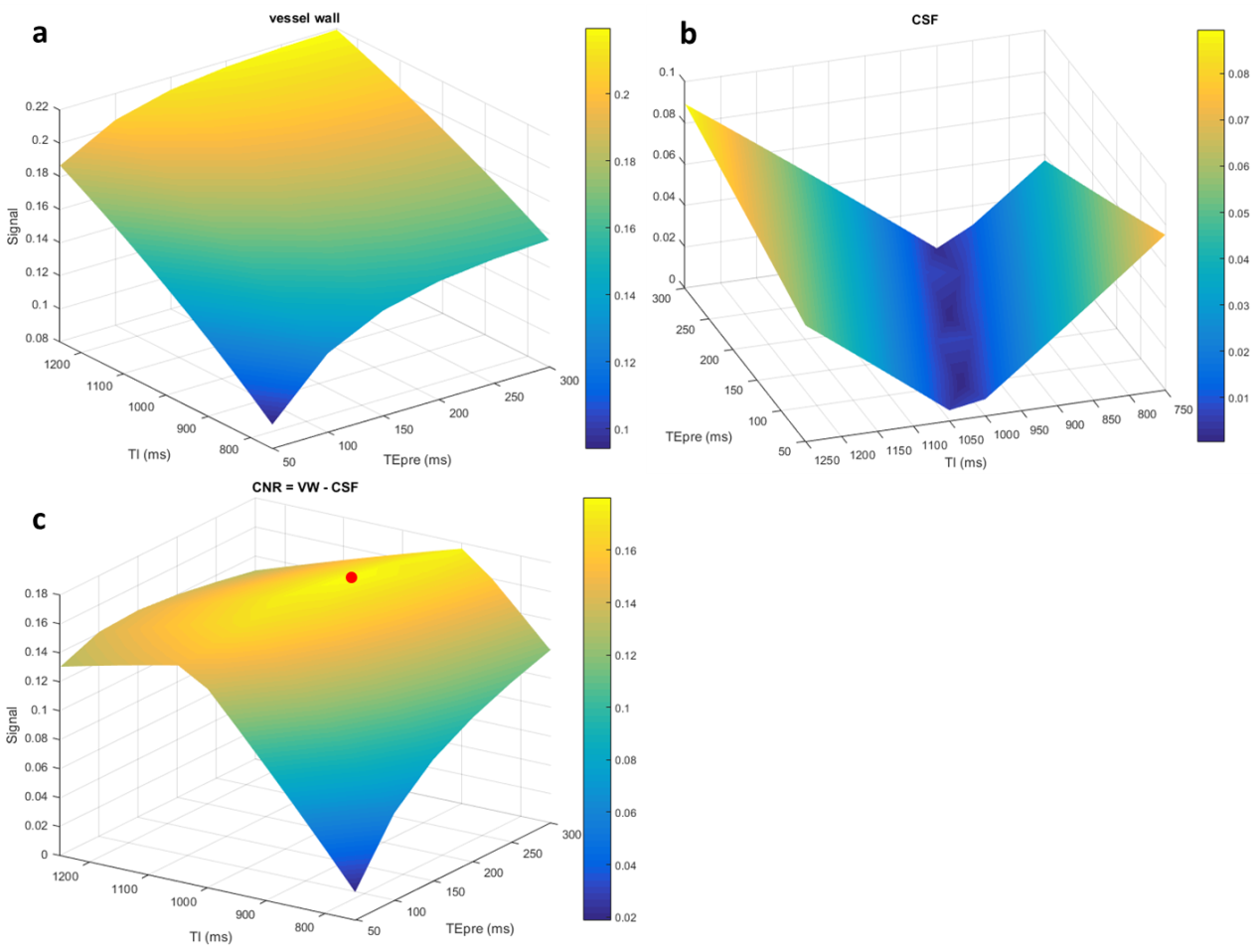


Supporting Figure 1: Simulated T_2_IR-SPACE signal intensity of (a) vessel wall, (b) CSF and (c) contrast between the vessel wall and CSF as a function of TI and TE_prep_.

**Optimization of T_2_w-SPACE.**

To optimize T2w-SPACE, we performed a series of T2w-SPACE images in a volunteer, with the parameters as mentioned in the manuscript, and the following TR values: TR=3000 ms, 2500 ms, 2000 ms, 1500 ms, and the acquisition times were: 14 min, 11 min 40 s, 9 min 20 s and 7 min, respectively. Supporting Figure 2 shows the long-axis images (upper row) and short-axis images (bottom row) of the four T2w-SPACE images. No clear optimum for the TR value/acquisition time was found, and the outer boundary of the intracranial vessel wall could not be differentiated from the surrounding CSF in all four image sets. Regardless of how to optimize the T2w-SPACE, the high CSF signal would deteriorate the intracranial vessel wall delineation. Since all four protocols cannot visualize the vessel wall, it is fairer to choose one (TR=2500 ms) with the same acquisition time (11 min 40 s) as T2IR-SPACE.


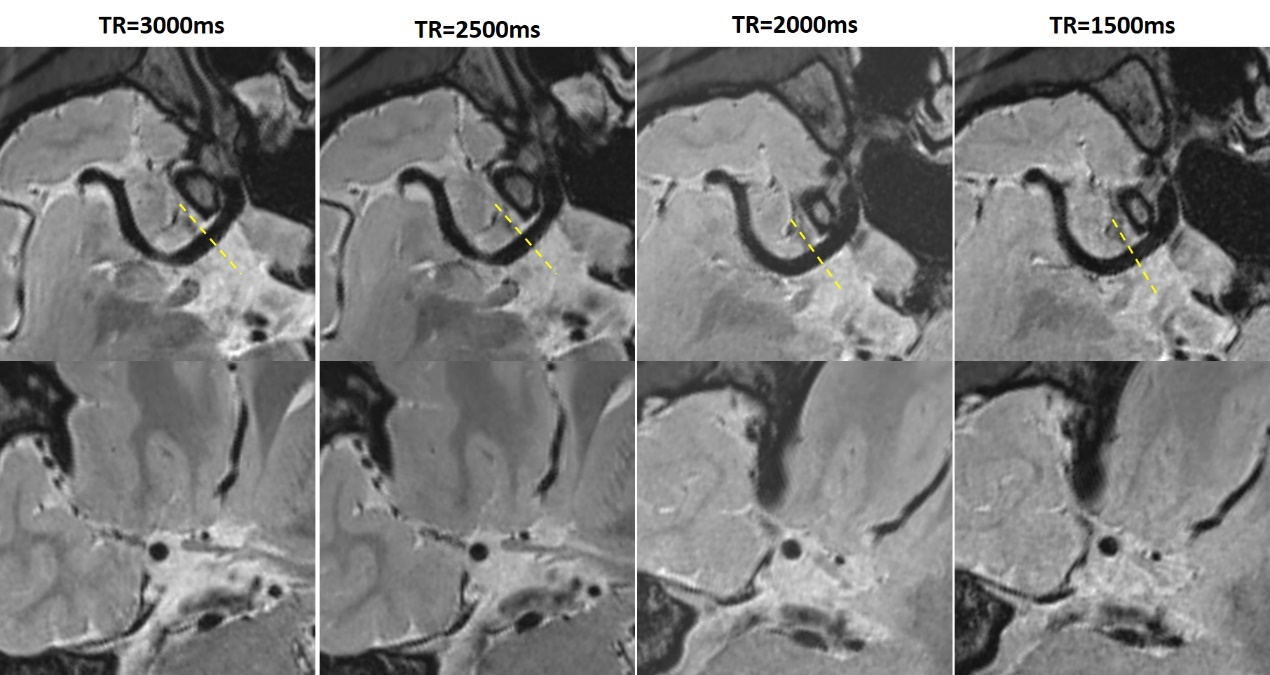


Supporting Figure 2: T2w-SPACE images with different TRs in a healthy subject.

**Simulation to study how much T1 weighting and T2 weighting are in T2IR-SPACE**

We performed simulations to study how much T1 weighting and T2 weighting are in T_2_IR-SPACE. First, we simulated the signal evolutions of two hypothetical tissues with different T1 values and the same T2 values. The T1 and T2 values for tissue 1 are 800 ms and 80 ms and 1000 ms and 80 ms for tissue 2. The simulation results are shown in Supporting Figure 3 (upper row). Tissue 1 with a short T1 value recovers faster than tissue 2 during the TI time interval (Fig 3a), and the signal difference between tissue 1 and tissue 2 is 0.0249 (the 23rd echo). Second, we simulated the signal evolutions of two tissues with different T2 values and the same T1 value: the same tissue 1 with T1=800 ms and T2=80 ms and tissue 3 with T1=800 ms and T2=100 ms. The simulation results are shown in Response Figure 3.3 (bottom row). Tissue 1 with a shorter T2 value decays faster than tissue 2 during the T2-prep time interval and echo train duration (Fig. 3c), and the signal difference between tissue 1 and tissue 3 is 0.0257 (the 23rd echo). From the simulation results, we can see that T2IR-SPACE is affected by T1 weighting next to the desired T2 weighting.


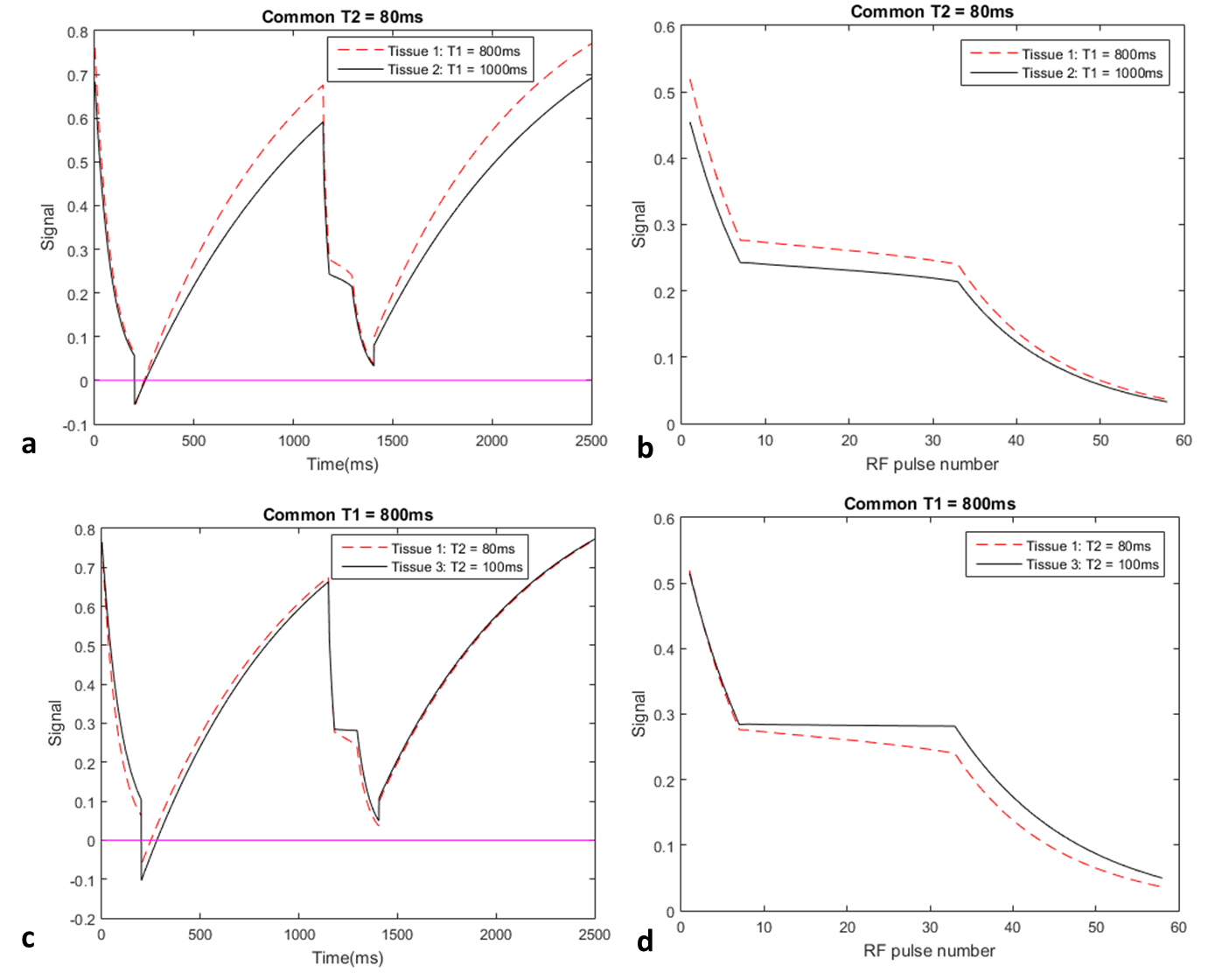


Supporting Figure 3: Simulation results of T2IR-SPACE from two hypothetical tissues with the same T2 value and different T1 values (a,b); the same T1 value and different T2 values (c,d).
